# Intergenerational transmission of childhood maltreatment and eating disorder behaviors: Shedding light on the mother-daughter dyad and grandmother-mother-daughter triad

**DOI:** 10.1192/j.eurpsy.2023.1107

**Published:** 2023-07-19

**Authors:** A. Talmon, N. Tsur

**Affiliations:** 1 Hebrew University, Jerusalem; 2 Tel Aviv University, Tel Aviv, Israel

## Abstract

**Introduction:**

Previous studies have established a relation between childhood maltreatment and eating disorder behaviors. However, this pattern of relations has not yet been studied within the nuclear family interactions.

**Objectives:**

The aim of this study was to examine a model illuminating the transgenerational mechanism underlying the association between childhood maltreatment and eating disorder behaviors.

**Methods:**

One-hundred-sixty-eight Israeli mothers and their young-adult-daughters (discovery sample) and 143 Israeli grandmother-mother-daughter triads (replication sample) filled out a battery of questionnaires assessing their history of childhood maltreatment and level of eating disorder behaviors.

**Results:**

Results of structural equation modeling (SEM) in the discovery sample indicated that mothers’ childhood maltreatment was associated with daughters’ childhood maltreatment and that mothers’ eating disorder behaviors were also associated with daughters’ eating disorder behaviors. In addition, for both mothers and daughters, childhood maltreatment was associated with eating disorder behaviors. Finally, an indirect effect was found in which the relation between mothers’ childhood maltreatment and daughters’ eating disorders was mediated by mothers’ eating disorders. Partial replication was observed; grandmothers’ childhood maltreatment was significantly associated with mothers’ childhood maltreatment. Grandmothers’ eating disorder behaviors were associated with mothers’ eating disorders and mothers’ eating disorders were associated with daughters’ eating disorders. Finally, an indirect effect was found in which the association between grandmothers’ eating disorders and daughters’ eating disorders were mediated by mothers’ eating disorders.

**Conclusions:**

These findings point to the significant contribution of the mother-daughter relationship in different aspects of the intergenerational transmission of both childhood maltreatment and eating disorder behaviors. These findings highlight the need to include a trauma-informed family-system approach in the treatment of eating disorders.

**Disclosure of Interest:**

None Declared

